# Embodied learning of a generative neural model for biological motion perception and inference

**DOI:** 10.3389/fncom.2015.00079

**Published:** 2015-07-06

**Authors:** Fabian Schrodt, Georg Layher, Heiko Neumann, Martin V. Butz

**Affiliations:** ^1^Cognitive Modeling, Department of Computer Science, University of TübingenTübingen, Germany; ^2^Institute of Neural Information Processing, Ulm UniversityUlm, Germany

**Keywords:** biological motion, correspondence problem, predictive coding, active inference, perspective-taking, embodiment, mirror neurons, neural networks

## Abstract

Although an action observation network and mirror neurons for understanding the actions and intentions of others have been under deep, interdisciplinary consideration over recent years, it remains largely unknown how the brain manages to map visually perceived biological motion of others onto its own motor system. This paper shows how such a mapping may be established, even if the biologically motion is visually perceived from a new vantage point. We introduce a learning artificial neural network model and evaluate it on full body motion tracking recordings. The model implements an embodied, predictive inference approach. It first learns to correlate and segment multimodal sensory streams of own bodily motion. In doing so, it becomes able to anticipate motion progression, to complete missing modal information, and to self-generate learned motion sequences. When biological motion of another person is observed, this self-knowledge is utilized to recognize similar motion patterns and predict their progress. Due to the relative encodings, the model shows strong robustness in recognition despite observing rather large varieties of body morphology and posture dynamics. By additionally equipping the model with the capability to rotate its visual frame of reference, it is able to deduce the visual perspective onto the observed person, establishing full consistency to the embodied self-motion encodings by means of active inference. In further support of its neuro-cognitive plausibility, we also model typical bistable perceptions when crucial depth information is missing. In sum, the introduced neural model proposes a solution to the problem of how the human brain may establish correspondence between observed bodily motion and its own motor system, thus offering a mechanism that supports the development of mirror neurons.

## 1. Introduction

Neuroscience has labeled a distributed network of brain regions that appears to be involved in action understanding and social cognition the *mirror neuron system* (Rizzolatti and Craighero, 2004, 2005; Iacoboni and Dapretto, 2006; Kilner et al., 2007; Iacoboni, 2009). Although the existence of mirror neurons in the human brain as well as their primary role for action understanding is still controversial (see e.g., the discussion after Lingnau et al., 2009), the existence of such a network and the inclusion of our own motor system in this network is generally accepted. However, it is still strongly disputed how this network may develop (Kilner and Lemon, 2013; Cook et al., 2014).

The mirror neuron system is believed to strongly interact with the Superior Temporal Sulcus (STS), forming an action observation network (Kilner et al., 2007). STS is particularly well-known for encoding biological motion patterns (Bruce et al., 1981; Perrett et al., 1985; Oram and Perrett, 1994) and has been considered as an important visual modality for the development of attributes linked with the mirror neuron system (Grossman et al., 2000; Gallese, 2001; Puce and Perrett, 2003; Ulloa and Pineda, 2007; Pavlova, 2012; Cook et al., 2014). The major portion of neurons in the posterior STS seems to encode viewer-centered representations of specific movements to the effect that their activation depends on the type of movement observed, as well as on the observer's current vantage point (Oram and Perrett, 1994; Perrett et al., 1985, 1989, 1991). Seeing that self-motions and motions of others are co-encoded in STS (Molenberghs et al., 2010), a correspondence problem arises (see Heyes, 2001; Dautenhahn and Nehaniv, 2002): How does observed biological motion, which is inevitably viewed from a perspective that does not correspond to a self-perceptual perspective, activate the same network of areas?

Besides the apparent integrative nature of STS, there is evidence for an integration of visual and proprioceptive information in monkeys in the parietal cortex (Graziano et al., 2000). Functional imaging suggests that visual and motor information are integrated in the human occipital-temporal cortex (Orlov et al., 2010). Yet how are motor and proprioceptive areas co-activated by visual perceptions of bodily motion even when they are observed from previously unexperienced vantage points? Considering human spatial abilities, several candidate mechanisms have been identified in psychometric studies (Lohman, 1979; McGee, 1979; Eliot and Smith, 1983; Carroll, 1993; Hegarty and Waller, 2004). Amongst them, *visuo-spatial perspective-taking* has been described as a progressive ability to adopt the spatial point of view of another person (Newcombe, 1989; Hegarty and Waller, 2004; Jackson et al., 2006).

We put forward an artificial generative neural model that offers a solution to the correspondence problem by employing a spatial perspective adaptation mechanism. The model is able to project visually perceived biological motion of others onto own action encodings, resulting in the co-activation of corresponding proprioceptive codes. Our model is embodied in the sense that it learns during simulated self-perception a generative model of biological motion by correlating corresponding relative motion in visual and proprioceptive pathways. By neurally deployed information preprocessing, the generative model achieves a fundamental invariance to several spatio-temporal transformations, including scale, translation, movement speed, and body morphology. Similar invariance properties have been observed in STS cells (Jellema and Perrett, 2006). Also in line with encodings of biological motion in STS, our learning algorithm is capable to encode visual motion redundantly in multiple orientations. Those view-dependent encodings form perceptual attractor states, which may be compared with attractors found for object recognition, in which mental rotations are involved (Palmer et al., 1981; Tarr and Pinker, 1989). In our case, an observed view of biological motion is seamlessly (mentally) rotated to the nearest orientation that was encoded during the training. In effect, also corresponding proprioceptive activities are coactivated, essentially simulating the observed motion with the own proprioceptive, embodied encodings.

The perceptual adaptation is essentially enabled by predictive coding schemes: The embodied, generative model of biological motion projects top-down its view-dependent expectation about the currently recognized motion. The mismatch between the expected and observed motion is compensated by a neural perspective-taking module: It continuously minimizes the error signal by rotating the whole visual percept and thereby essentially establishes the correspondence between different perspectives. Naturally, having encoded a number of different views of the same biological motion improves the performance of this process.

In sum, we show that the correspondence problem can indeed be solved by an embodied, generative neural network model that is able to adapt to the individual perspectives of others. More specifically, we show how bodily motions perceived visually could map to proprioceptive encodings regardless whether observed or performed. Combined with other mechanisms, we suggest that the model offers a solution to how a social, mirror neuron network may develop and how this network may be activated given visual motion information only.

We detail the neural architecture for learning, recognition, and inference of biological motion in the next section. The model is evaluated in several experimental setups in Section 3, showing robust learning of one or multiple views of biological motion and the flexible adaptation of the internal perspective upon the presentation of novel views. Next, we discuss related modeling approaches in Section 4. Finally, we summarize the results, draw conclusions, and sketch-out future research perspectives in Section 5.

## 2. Generative neural network model description

Given streams of time series data from neurally processed visual and proprioceptive pathways, the generative neural model learns to (1) spatially correlate the data by predictive inference principles, (2) segment the data into motion patterns, and (3) temporally correlate the data by learning predictive transition probabilities. In the following we detail how the model neurally processes the data streams and how it learns to spatially and temporally correlate and segment them. Moreover, the adaptation of the internal perspective onto the visual information is detailed as well as the continuous minimization of predictive errors by neural activity adaptations.

**Figure 1:**
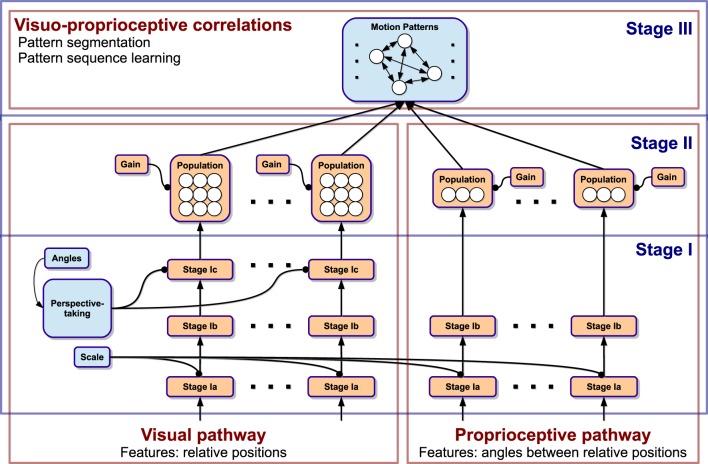
**Diagram of the 3-stage neural network model and the processing paths**. The model is driven by visual and proprioceptive features. In Stage I, generalization over spatio-temporal transformations is achieved. Stage II converts the coding scheme to population coding. Stage III accounts for segmentation of the driving data into motion patterns and learning of the temporal sequence of patterns.

The model essentially consists of a three-stage neural processing cascade illustrated in Figure 1. Stage I preprocesses relative information from vision and proprioception to account for multiple invariances in translation, scale, speed, body morphology, and spatial orientation. This is achieved by transforming the data into the velocity domain, normalization, and self-supervised perspective-taking. Stage II converts the neural coding scheme into population encodings, which account for directional motion signals. Then, a common visuo-proprioceptive domain is created by incorporating all visual and proprioceptive populations into a multimodal feature pool. Stage III implements spatio-temporal segmentation in this multimodal domain given a continuous stream of sensory signals, which enables the predictive encoding of biological motion patterns and sequences.

Technically, Stage III is akin to an extended, neural noise-based adaptive resonance model (Grossberg, 1976). On the one hand, it learns a set of motion patterns representing recurring visuo-proprioceptive correlations, which serve both as recognizers as well as predictors of currently observed motion. On the other hand, each recognized pattern learns a lateral, probabilistic influence on the subsequent recognition of other patterns, effectively encoding whole sequences of recognized patterns. As a result, the model privileges the recognition of familiar motion pattern sequences when unfamiliar stimuli are presented. Moreover, this enables the simulation of the same sequences when no stimulus is present.

In the following, we detail the three neural processing stages and the involved information processing and adaptation techniques.

### 2.1. Stage I - feature processing

Our embodied model approach assumes that knowledge about the own body dynamics is useful for understanding bodily motion of others. When observing another person's motion, however, it is necessary to generalize self-generated motion to similar, observed biological motion generated by others. An observed person will most likely exhibit a slightly different body morphology and, even more importantly, will be perceived from a different vantage point, which results in a translation and typically a rotation with respect to the observer's frame of reference. Also the speed and accelerations of the other person's motion dynamics will typically differ.

The neural information preprocessing in Stage I generates normalized relative directional motion signals, yielding signals that are generally invariant to scale, speed, body morphology, and translation. The differences in orientation are eliminated by a self-supervised, online rotation of perceived motion in the three-dimensional, visual pathway. This perspective-taking mechanism basically enables the establishment of correspondences between executed and observed motion. Moreover, it enables the derivation of the orientation of the observed person relative to ones own perspective, rather than just encoding biological motion view-independently.

**Figure 2:**
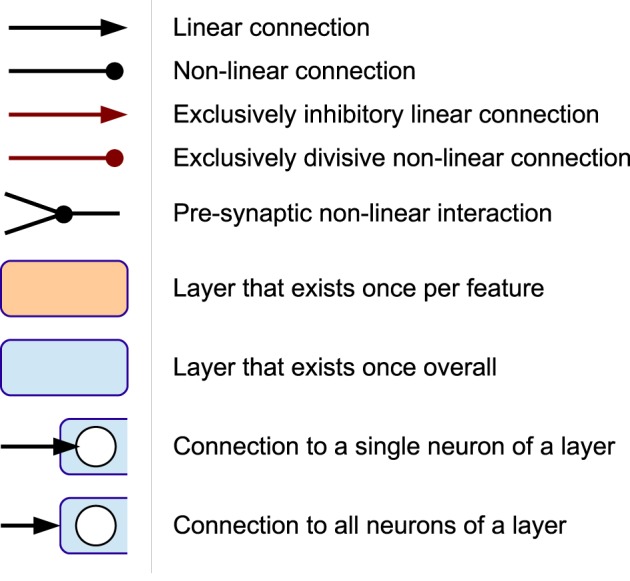
**Legend for the neural connectivity schemes**.

**Figure 3:**
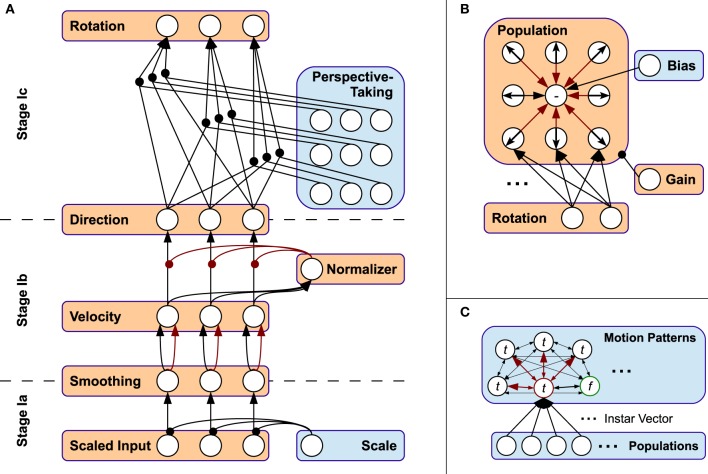
**Connectivity schemes of Stages I-III. (A)** Neural connectivity in Stage I. **(B)** Population coding, simplified for 2D. **(C)** Pattern layer with lateral inhibition. t: trained pattern, f: free pattern (green circle). Red circle: current winner pattern.

The neural connectivity that results in this information preprocessing is shown in **Figure 3A**. A legend for this and the following connectivity diagrams is shown in Figure 2. The model's neural activity is driven bottom-up by sets of visual and proprioceptive features. A visual input feature is defined by a relative position between two bodily landmarks; for example, the hand position relative to (minus) the elbow position or relative to the center of the body. Proprioceptive inputs to the model are defined by angles between relative positions^1^. We are assuming that depth information is available and that the identification of respective body parts has taken place beforehand. Thus, especially the selection of relative bodily features is predefined as is the assignment of the selected features to their respective neural network inputs. The choice and number of bodily features is not particularly relevant for the functionality of our model, though, as long as the information is sufficiently expressive. In the experiments, we also show that depth information is not necessary for successful biological motion recognition. The automatic selection of relative bodily features and the automatic assignment of these features to the respective neural network inputs, however, remains a challenge for future work.

In sum, the input to the network is driven by a number of both, relative 3D positions in the visual pathway and 1D angles in the proprioceptive pathway, each commonly denoted by a vector ${\vec{x}}_{n}$.

#### 2.1.1. General neural and synaptic building blocks

We formalize the general functionality of our neural network by nesting potentially recurrent and non-linear functions (italic in the following) of multiple action potentials into both the synaptic and axonic responses of neurons. The output *o_j_*(*t*) of a neuron indexed *j* in terms of the afferent firing rate is determined by the product of an *activation function f_j_*(net_*j*_(*t*)) of the neuron's net input net_*j*_(*t*), with a *gain control function a_j_*(*t*):

(1) $$o_{j}(t)=a_{j}(t)\cdot f_{j}({\text{net}}_{j}(t))\;.$$

The net input net_*j*_(*t*) to neuron *j* sums up all synaptic inputs to the neuron. Each synaptic input is determined by a *pre-synaptic process function s_Ij_*(…) that consolidates interactions between pre-synaptic cells connected via the same synapse, and a synaptic transfer factor *w_Ij_*(*t*) that describes the local efficacy or weighting of an axon-postsynaptic transmission. It is either a constant factor or a function of time in case it is adapted by a learning rule. In effect, the input to a neuron *j* via a single synapse is a weighted function of the output of preceding neurons that are indexed in the set *I*. In sum, this leads to the net input

(2) $${\text{net}}_{j}(t)=\sum_{I\in A_{j}}s_{Ij}(o_{I_{1}}(t),o_{I_{2}}(t),\ldots )\cdot w_{Ij}(t)\;,$$

where *A_j_* denotes the superset of all sets of neurons synaptically connected to neuron *j* and thus afferently contributing to its input. Each specific pre-synaptic function *s_Ij_*(…) processes several neural outputs in a systematic, potentially non-linear fashion. For example, a pre-synaptic process function

(3) $$s_{Ij}(o_{I_{1}}(t),o_{I_{2}}(t))=o_{I_{1}}(t)\cdot o_{I_{2}}(t)$$

implements a gain-field multiplication (Andersen et al., 1985) of the output of the neurons *o*_*I*_1__(*t*) and *o*_*I*_2__(*t*). However, other functions are possible in our model depending on an abstract extra-cellular connectivity. Also, a single neuron may determine its net input via a number of different pre-synaptic process functions.

Analogously to synaptic inputs, the modulation of a neuron's output by its gain control function is given by

(4) $$a_{j}(t)=\prod_{I\in \Omega_{j}}s_{Ij}(o_{I_{1}}(t),o_{I_{2}}(t),\ldots )\cdot w_{Ij}(t)\;,$$

where Ω_*j*_ denotes the superset of all sets of neurons postsynaptically connected to neuron *j* and thus controlling its efferent signal. Here, we assume that postsynaptic modulation of a neuron has a multiplicative influence on the neuron's firing rate. In this way, for example, shunting inhibition (Eccles, 1964) can be implemented. The rules for gradient descent by backpropagation over pre- and post-synaptic connections can be derived for the above formalism. Unless declared otherwise, activation functions of the model's neurons are linear, synaptic weightings and gain control functions are neutral, and pre-synaptic process functions at synapses are passing a single preceding neuron's output.

#### 2.1.2. Ia—scaling and smoothing

The overall purpose of processing cascade Stage I is to encode the motion direction of each relative feature considered. However, it may occur that a feature does not have a velocity and thus no direction, or a velocity with a magnitude below a certain threshold and thus a direction with minor validity considering potential noise in the data. To parameterize this threshold, Stage Ia applies a constant scaling factor α to every visual or proprioceptive input ${\vec{x}}_{n}$using the gain control modulation *a_i_*(*t*) of all input neurons of the network, here indexed by *i*:

(5) $${\text{net}}_{i}(t)\;=x_{i}(t)\;,$$

(6) $$a_{i}(t)\;=\alpha .$$

This later on decides whether a local feature (e.g., the position of the hand) is considered as static or dynamic in a global movement (see normalization in Stage Ib). Furthermore, a simple way to avoid strong changes in the calculated direction is exponential smoothing with an update-factor λ (cf. Sutton and Barto, 1981), which is implemented via inert, pairwise connections from the input layer *i* to the scaled and smoothed layer indexed by *j* (which is equivalent to the smoothing layer between Stage Ia and Stage Ib indicated in Figure 3A):

(7) $$s_{ij}(t)=s_{ij}(t\text{​}-\text{​}1)\cdot \lambda +o_{i}(t)\cdot (1\text{​}-\text{​}\lambda ).$$

#### 2.1.3. Ib—normalized directional velocity

Stage Ib calculates the normalized direction of the movement of a relative feature. This means that only the motion direction is regarded, while the magnitude of velocity is generalized over^2^. In this step, the model gains invariance to several spatio-temporal transformations in perceived biological motion: Since the positions considered are relative to each other rather than relative to a global reference point, a general invariance to translation is accomplished. A specific, basic body structure knowledge is provided, according to which the relativities are selected. Furthermore, the *direction* of movements generalizes over the magnitude of relative velocities, resulting in a spatial scale invariance and an additional temporal invariance to the timescale and overall speed of the observed movements. We apply this feature processing to each single, relative feature that is considered. Since this analogously results in scale invariance for each single positional relativity, that is any limb length when features are provided accordingly, the model is able to completely generalize over the body morphology of an observed actor (cf. Schrodt and Butz, 2014).

Stage Ib consists of three layers, whose neurons are indexed *i*, *j*, and *k*, where *i* now corresponds to the neurons' indices of the scaled and smoothed output layer of Stage Ia, and *j* corresponds to the velocity-layer of a feature indicated in Figure 3A. First, the velocity calculated in layer *j* results from pre-synaptic processes in a pairwise connection scheme with layer *i*. These pre-synaptic process functions are implemented by time-delayed inhibition, basically calculating the temporal difference of the preceding neuron's activation:

(8) $$s_{ij}(t)=o_{i}(t)-o_{i}(t\text{​}-\text{​}1),$$

Secondly, a real-time normalization is performed by pairwise connections to the neurons of the stage's output layer indexed by *k*. Yet, each connection modulates the output of a velocity neuron by a single, feature-specific normalizer neuron with index *l*. To normalize the activities in layer *k* to length 1, its gain factor is determined by the inverse length of the output vector of layer *j*. This can be denoted by

(9) $$s_{\{j,l\}k}(o_{j}(t),o_{l}(t))\;=o_{j}(t)\cdot o_{l}(t),$$

(10) $$f_{l}({\text{net}}_{l}(t))\;=\min\left(\frac{1}{\sqrt{{\text{net}}_{l}(t)}},1\right)\in (0,1],$$

(11) $$s_{jl}(o_{j}(t))\;=o_{j}{\left(t\right)}^{2}.$$

By Equation (10), it can be seen that each feature-specific normalizer neuron *l* is limited to an *inhibition* of the input to layer *k*, which means that only features with a velocity magnitude > 1 are normalized to length 1. This is the case, if the length of a hypothetical velocity vector of a feature fed into the model is ≥ 1/α (see Equation 6). Otherwise, the length of the resulting direction in layer *k* will be < 1. Although this means that the model loses a bit of invariance to scale, translation, and speed, this mechanism is important for the model's robustness to small motion signals, as explained in detail in Section 2.2.

#### 2.1.4. Ic—perspective-taking

Interstage Ic accounts for the orientation invariance and thus—together with the properties mentioned beforehand—for affine invariance of the model's three-dimensional visual perceptual system. Since one-dimensional features are invariant to rotation in general, a rotation mechanism in the proprioceptive pathway is not necessary and thus not applied. Visual orientation invariance is achieved by applying the same adequate rotation to every visual, normalized velocity from Stage Ib. The rotation is realized by a perspective-taking module, whose linear output neurons directly map the elements of a rotation matrix originating from the Euler rotation^3^ sequence z-y-x. Then, the neural connectivity between the input and output layers of Stage Ic reflects a gain-field-like modulation of a full connection between input and output layer of this sub-stage, as shown in Figure 3A. Note that this systematic, triple-wise pre-synaptic connection scheme is equivalent to a matrix-vector multiplication.

**Figure 4:**
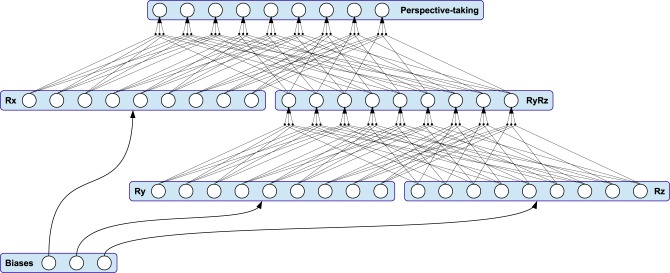
**The connectivity pattern of the perspective-taking module is equivalent to multiple matrix-matrix multiplications**. The module is driven by three adaptive thresholds, each representing an Euler rotation angle applied to the visual pathway.

The neural perspective-taking module is exemplified in Figure 4. It consists of three sub-modules *R_x_*, *R_y_* and *R_z_*—each representing an axis-specific rotation matrix—and an intermediate module. Accordingly, the matrices of activation functions for the three sub-modules are

(12) $$\begin{array}{l} R_{x}=\left(\begin{array}{ccc} 1 & 0 & 0\\ 0 & \cos\mu_{x} & -\sin\mu_{x}\\ 0 & \sin\mu_{x} & \text{cos}\mu_{x} \end{array}\right)\\ R_{y}=\left(\begin{array}{ccc} \cos\mu_{y} & 0 & \sin\mu_{y}\\ 0 & 1 & 0\\ -\sin\mu_{y} & 0 & \cos\mu_{y} \end{array}\right)\\ R_{z}=\left(\begin{array}{ccc} \cos\mu_{z} & -\sin\mu_{z} & 0\\ \sin\mu_{z} & \cos\mu_{z} & 0\\ 0 & 0 & 1 \end{array}\right)\;. \end{array}$$

Here, the respective pre-synaptic connection scheme is completely equivalent to encapsulated matrix-matrix multiplications, resulting in the activation function matrix *R*_μ_ = *R_x_R_y_R_z_* of the module's output. Thus, this output is again a rotation matrix, restricting all transformations of motion features performed in instances of this stage to the same, length-preserving rotation. The magnitude of rotation about each axis is determined by three adaptive bias neurons with output μ_*x*_, μ_*y*_, and μ_*z*_: As shown in Figure 4, the bias neurons feed every neuron in their respective sub-module, while their output is equivalent to a rotation angle about an Euclidean axis.

To solve the correspondence problem by taking the perspective of an observed actor, the rotation of biological motion observed from an unknown vantage point is ideally equivalent to the rotation to the next perspective-attractor seen during the embodied training. However, the model has no explicit knowledge about the correct rotation. Rather, it is adapting the rotation dynamically according to a top-down propagated error signal gathered from the predictions of multimodal patters that encode visuo-proprioceptive correlations, as described in Section 2.3. Resulting from the connection scheme, top-down propagated feedback signals are merged from all visual feature paths into the perspective-taking module and are processed in a way that restricts the perceptual adaptation to an angular momentum in a three-dimensional Euclidean space. This is realized by adapting the biases of the module according to on-line gradient descent with learning rate η_μ_ and momentum *m*_μ_.

From a psychological perspective, this rotation can be considered to perform visuo-spatial perspective-taking, because it internally rotates the visual perception of another person (and as a matter of modeling, also its environment) into a previously learned frame of reference. Further, by reading out the activation of the adaptive bias neurons after adaptation, one can directly determine the orientation of an observed actor relative to the own simulated frame of reference. Technically, this adaptation can be compared to (Tani, 2003; Sugita et al., 2011), where batch adaptation of bias neurons was used to achieve different behavioral primitives. However, the simultaneous and steady adaptation of the orientation of perceived biological motion we use allows the derivation of the path of rotation toward an error-minimal perspective. This is in compliance with psychometric studies on mental rotation (Shepard and Metzler, 1971).

We are aware that in neurobiological terms classical backpropagation of a supervised learning signal and parameter optimization through gradient descent may not be considered the most plausible approach. However, our idea differs in the way that the learning signal emerges completely without exogenous teaching, but rather from motion patterns that have been learned in an unsupervised manner. In this way, this self-supervised backpropagation can be compared to feedback connections that implement a predictive encoding paradigm, while gradient descent ensures the convergence to a minimum in free energy, or in other words, to perceptual attractor states. Here, those attractor states are equivalent to view-dependent encodings of biological motion.

In sum, stage I provides autonomously rotated, normalized directions of visuo-proprioceptive motions. It accounts for affine invariance in biological motion perception. The information preprocessed in such a way serves as input to stage II, where population coding is performed.

### 2.2. Stage II—population coding

Stage II accounts for population coding of the individual features under consideration of the overall length of activations in the populations, which serves as preparation for the segmentation and predictive coding applied in stage III. This step is shown as connection graph in Figure 3B for a single feature.

The normalized motion direction of a feature—encoded by a layer with neurons indexed by *i*—can be converted into a symmetric population of direction-selective neurons—encoded by a layer with neurons indexed by *j*—by full connection via a directional weighting matrix *W_ij_*: This weighing matrix is set up in a combinatorial fashion, as every single dimension of the *D_n_*-dimensional motion direction of the *n*th feature may be positive, neutral, or negative. This results in 3^*D*_*n*_^ − 1 possible combinations of motion directions. For instance, in a 2D example this would result in

(13) $$W_{ij}=\left(\begin{array}{cc} 0 & 1\\ 1/\sqrt{2} & 1/\sqrt{2}\\ 1 & 0\\ 1/\sqrt{2} & -1/\sqrt{2}\\ 0 & -1\\ -1/\sqrt{2} & -1/\sqrt{2}\\ -1 & 0\\ -1/\sqrt{2} & 1/\sqrt{2} \end{array}\right)\cdot \beta_{n}\;,$$

where each row of the matrix describes a synaptic weight vector ${\vec{w}}_{Ij}$ of a population neuron by means of a normalized direction that represents the tuning of the neuron to the directional motion input. The tuning of the population neurons is equally distributed and overlapping to cover the whole directional space. β_*n*_ denotes a specific scaling factor that results in a normalization of length 1 of the concatenation of all feature populations (see below). In summary, this mechanism provides a population of neurons for each feature of sensory processing, which is either sensitive to directional motion in a body-relative limb position (26 neurons for each position) or sensitive to directional motion in angles between limbs (2 neurons for each angle).

Additionally, each population provides a single neuron with index *s* that is active only when the feature velocity is very small and thus normalized to a magnitude < 1 (cf. Equation 10): Its activation is calculated in a way such that a population's response vector including neuron *s* warrants a specific length *L*, even if the actual length of the direction-selective neurons is *l* < *L* due to an insufficient normalization in Stage Ib. This can be performed by lateral inhibitory connections from all 3^*D*^ − 1 direction sensitive neurons *j* plus a single bias neuron *b* connected to neuron *s*:

(14) $$f_{s}(t)=\sqrt{{\text{net}}_{s}(t)}\;,$$

(15) $$s_{js}(t)=-o_{j}{\left(t\right)}^{2}\;,$$

(16) $$s_{bs}(t)=o_{b}(t)\;,$$

(17) $$o_{b}(t)=L^{2}\;,$$

such that $o_{s}(t)=\sqrt{L^{2}-l^{2}}$. As a result, the total length of the population including neuron *s* is equal to *L* in all possible cases. The desired length *L* of each feature population can be determined by $1/\sqrt{N+M}$, where *N* denotes the number of visual features, and *M* denotes the number of proprioceptive features processed. Together with

(18) $$\beta_{n}=L\cdot \sqrt{\frac{D_{n}}{3^{D_{n}}-1}}\;,$$

where *D_n_* denotes the dimensionality of feature *n*, it is assured that the length of the concatenation of all feature populations is 1. Consequent normalization of the data is an important prerequisite for the pattern learning applied in Stage III.

To distinguish features without velocity from features that are not observable at the present time, we add an exogenous gain factor *g_n_*(*t*) as gain control to every neuron *j* of a feature specific population *n*, such that

(19) $$a_{j}(t)=g_{n}(t)\in \{0,1\}\;\forall j\;.$$

While initially, this gain factor is 1, it is set to 0 if a feature is considered unavailable.

Both, the direction sensitive neurons and the neuron sensitive to no velocity and their connectivity are shown in Figure 3C. The activations provided by the concatenation of all population neurons serves as input to stage III, where spatio-temporal learning of motion patterns is applied to infer predictions about the progress of observed movements.

### 2.3. Stage III—segmentation and predictive coding

While Stage I and Stage II essentially pre-process incoming information extracting relative motion signals by means of a pre-wired architecture, Stage III is a network with adaptive connectivity that performs unsupervised segmentation of the driving data into sets of motion patterns and that learns about the temporal sequence of the developing patterns. Considering the input fed into the model and the processing described, each pattern represents a recurring visuo-proprioceptive correlation in a high-dimensional, highly invariant, directional motion space. The embodied learning procedure entails that at first, self-induced biological motion is observed from one or multiple, rather arbitrary egocentric views (without error-driven adaptation of the visual frame of reference). Patterns are recruited probabilistically when sufficiently new and unexpected data is perceived, which we describe in Section 2.3.1. For temporal sequence learning, which we describe in Section 2.3.2, each pattern develops asymmetrical lateral connections, which encode and privilege possible pattern sequences by exploiting neural noise paradigms.

In this way, while training, pattern sequences that repeatedly occurred are preferably recognized and developed further, which largely improves the self-supervised distinction of different movements in terms of patterns unambiguously responding to specific views and movements. After training, those patterns serve both as recognizer as well as predictor of currently observed motion. This means that the view-dependence of observed motion can be resolved by minimizing the error in the prediction after a pattern has been recognized. Again, pattern recognition is highly improved by learning about likely pattern sequences, and can be enhanced further by providing basically view-point invariant angular motion features.

#### 2.3.1. Spatial pattern learning

The unsupervised clustering of activations given by the concatenation of all motion-encoding population neurons from Stage II (in the following indexed by *i* ϵ *I*) by means of a number of motion pattern responsive neurons (indexed by *j*) is achieved via a full connection in-between the layers. Each pattern neuron *j* is responsible for a specific, local part in the high-dimensional space of possible population activities, which is encoded in its instar weight vector denoted by ${\vec{w}}_{Ij}$.

Because the population activation space to segment can be arbitrarily high-dimensional and complex, we bootstrap both the number of patterns and their initial response from scratch without prior knowledge about the final distribution. First, this means that instead of initializing the weights randomly, they are initialized with ${\vec{w}}_{Ij}(t\text{​}=\text{​}0)=\vec{0}$. Secondly, the pattern layer is growing dynamically.

The pattern neurons feature a neural noise-based activation function, which distinguishes two types of patterns by the length of their instar weight vector:

(20) $$f_{j}({\text{net}}_{j}(t))=\{\begin{array}{ll} \mathbb{C}(\gamma ,{\text{net}}_{j}(t)) & \text{if }j\text{ is a trained pattern}:||{\vec{w}}_{Ij}(t)||>r\\ \mathbb{C}(\gamma ,\theta ) & \text{if }j\text{ is a free pattern}:||{\vec{w}}_{Ij}(t)||\leq r \end{array},$$

where ℂ(γ, *x*) denotes a Cauchy-distributed random variable with scaling γ and mean *x*. By *r*, we introduce the minimal length of the instar vector to a pattern, below which a pattern neuron is considered *free* in the sense that it can be acquired to encode new, previously unseen data. A free pattern does not respond to input, instead, its expected activation is parameterized by the constant θ. All patterns that feature an instar length above *r* are considered *trained*, in the sense that they are responding to the sensory signals.

By adapting the instar weight vector ${\vec{w}}_{Ik}(t)$ of a pattern *k* to the input activation ${\vec{o}}_{I}(t)$ fed forward by the populations, the pattern neuron increases its tuning to the respective constellation of positional and angular directional motion. Adaptation is achieved by the associative learning rule

(21) $$1/\eta \cdot \partial w_{ik}(t)/\partial t\;=\Delta w_{ik}(t)=o_{i}(t)-w_{ik}(t)\;,$$

where η denotes the learning rate for encoded motion patterns. We define η > *r*, such that, if *k* is a free pattern, it is converted into a trained pattern once it is adapted a single time. Starting with only one single free pattern, a new free pattern is created and connected accordingly as soon as the former free pattern is recruited, that is when $||{\vec{w}}_{Ij}||>r\;\forall j$, thus growing a new free pattern neuron on demand.

From the pre-synaptic process function of pattern neurons

(22) $$s_{ij}(o_{i}(t))=\frac{o_{i}(t)}{\max(||{\vec{w}}_{Ij}(t)||,r)}\;,$$

where we assume that neural excitability decreases proportional to the overall synaptic strength, it follows that the input to a trained pattern neuron *j* is determined by the angle between the observed pattern ${\vec{o}}_{I}(t)$ and the pattern stored in the instar weight vector ${\vec{w}}_{Ij}$(*t*), since both are normalized unit vectors:

(23) $${\text{net}}_{j}(t)=\sum_{i\in I}w_{ij}(t)\cdot s_{ij}(o_{i}(t))\;,$$

(24) $$=\cos(∡({\vec{w}}_{Ij}(t),{\vec{o}}_{I}(t)))\;.$$

This property ensures that the pattern neuron with the smallest angular distance to a given input vector is (most likely) the most active, which we call the *winner pattern k*. Only the winner pattern *k* is adapted to the currently propagated activation (winner-takes-all learning). If the most active pattern is the free pattern, it is recruited to represent the current data. Although trained instar vectors are normalized by the presynaptic process function above, their length still has an influence on the rate of adaptation of a pattern's directional tuning.

The neural noise activation function in combination with winner-takes-all learning accounts for (1) probabilistic updates of one of several similarly close trained patterns, (2) a deterministic influence on when to train a new pattern, determined by the threshold parameter θ, and (3) a probabilistic influence on when to recruit a new pattern, determined by γ. By parameterizing the probabilistic and deterministic influence on pattern recruitment accordingly, the segmentation paradigm can account for a specific degree of generalization and robustness against noise in the driving data. Since the random variable is Cauchy-distributed, the probability that a free pattern *f* has a higher activation than the best matching trained pattern *g*, and thus will be the next winner *k* that is trained on the observed data, can be determined in closed form depending on the input to neuron *g* (see Supplementary Material for the derivation):

(25) $$p(o_{f}(t)\geq o_{g}(t))\;=\;1/2+\;1/\pi \cdot \arctan\left(\frac{\theta -{\text{net}}_{g}(t)}{2\gamma }\right)\;.$$

**Figure 5:**
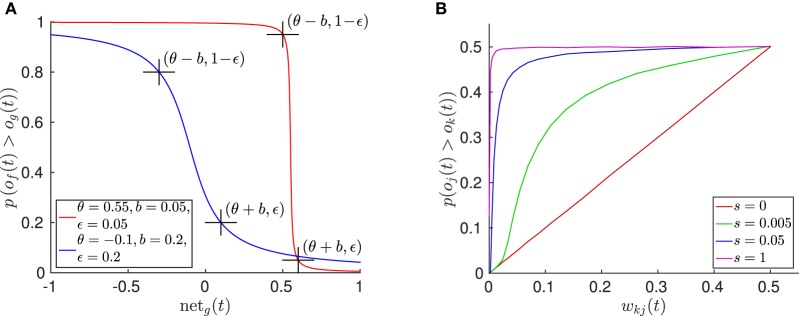
**Properties of the spatial and temporal pattern learning algorithm. (A)** Plot of the cumulative pattern recruitment probability for two exemplar function parameterizations. Here, *o_g_*(*t*) denotes the activation of the best matching, trained pattern, and *o_f_*(*t*) denotes the activation of the free pattern. **(B)** Plot of the effect caused by temporal pattern learning, yielding temporally predictive, lateral inhibition between pattern neurons. Here the inhibition effect of pattern neuron *k* on pattern neuron *j* is illustrated, which is determined by *w_kj_*. The relative pattern winning probability of *j* over *k* depends on the signal strength *s*, given that both neurons are randomly activated with equal strength on average. The plot shows the result of 500 k randomly sampled trials per measuring point of *w_kj_* and *s*, in which both neural activations were set uniformly randomly to *o_k_*(*t*) ϵ [−1, 1] · *s* and *o_j_*(*t*) ϵ [−1, 1] · *s*. The result shows that with increasing signal strength the influence of the lateral bias decreases, while the lateral inhibition nearly fully determines the transition probability given a very weak signal.

This cumulative distribution function is plotted in Figure 5A, showing the resulting sigmoidal function, which is symmetric around the pattern recruitment threshold θ, where (θ) is the angular mismatch in rad between the instar vector ${\vec{w}}_{{}_{Ig}}$ of the best matching pattern *g* and the actually observed stimulus ${\vec{o}}_{I}$, for which the probability to recruit a new pattern is 0.5. The scaling of the pattern noise γ reflects the fuzziness of this threshold and can be parameterized by choosing a probabilistic recruitment remainder ϵ and a breadth *b*, for which

(26) $$p(o_{f}(t)\geq o_{g}(t)|{\text{net}}_{g}(t)\text{​}=\text{​}\theta +b)=\epsilon ,\;\;\epsilon \leq 0.5,\;\;b>0\;,\;$$

or analogously

(27) $$p(o_{f}(t)\geq o_{g}(t)\;|{\text{net}}_{g}(t)\text{​}=\text{​}\theta -b)=1\text{​}-\text{​}\epsilon \;,$$

such that there is a low probability ϵ to recruit a new pattern if the best matching trained pattern's input is θ + *b*, or a high probability 1 − ϵ to recruit a new pattern if the input is θ − *b*, respectively. From this it follows that (see Supplementary Material)

(28) $$\gamma =\frac{\tan(\epsilon \pi )\cdot b}{2}\;.$$

#### 2.3.2. Temporal pattern learning

A further characteristic of the neural noise-based activation function of pattern neurons is that by asymmetric lateral inhibitory biasing, implicit winner sequences can be encoded. Given that the pattern layer is fully, reciprocally connected without self-connections, the winner neuron *k*(*t* − 1) determined in the last time step is able to inhibit all other neurons *j* (except the free pattern), that is, all potential successors in the sequence of winning patterns, such that the probability for another pattern to have a larger activation than the last winner *p*(*o_j_*(*t*) > *o*_*k*(*t*−1)_(*t*)) is equal to the lateral weight *w*_*k*(*t*−1)*j*_. Given that the lateral weights approximate this independent probability while learning, the inhibition implicitly establishes the *dependent* winning probability *p*(*o_i_*(*t*) > max_*j*≠*i*_(*o_j_*(*t*))) for a new pattern neuron *i* winning in the current time step. This lateral inhibition can thus be considered a time-dependent prediction of the next winner pattern.

Since winner pattern transitions should not be driven purely by lateral inhibition, but mainly by stimulus, we make two assumptions for the derivation: First, we assume that currently no sensory input is given. Secondly, we limit the lateral inhibition to the interval (−1, 0] by applying a hyperbolic tangent for signal transformation. This results in the laterally inhibiting pre-synaptic process function

(29) $$s_{kj}(w_{kj}(t))=\tanh\left(\frac{-2\gamma }{\tan\left(w_{kj}(t)\pi \right)}\right)\in (-1,0]\;\text{for}\;w_{kj}(t)\leq 0.5\;,$$

which causes that the probability for the activation of pattern *j* to be greater than the activation of the winner pattern *k* is approximately *w_kj_*, if there is no sensory input (see Supplementary Material for the derivation). Note that the lateral inhibition is added to the input to each pattern neuron (cf. Equation 23). Accordingly, the influence of this lateral inhibition on the actual probability *p*(*o_j_*(*t*) > *o_k_*(*t*)) when the network is driven by sensory signals depends on the total signal strength, which is determined by the number of currently observable features (see Equation 19). Figure 5B illustrates this interaction.

The lateral weights *w_kj_* are initialized with 0.5, such that no inhibition occurs. While training the network on biological motion, the weights outgoing the last winner *k*(*t* − 1) are adapted to a stochastically determined expectancy of *p*(*o_j_*(*t*) > *o_k_*(*t*)) by batch learning, when a transition event in the current winner pattern is detected:

(30) $$\frac{\partial w_{kj}(t)}{\partial t}=\{\begin{array}{ll} \;\eta_{l}/T\cdot \sum_{\tau =t-T}^{t}\left(\;1/2+\;1/\pi \cdot \arctan\left(\frac{{\text{net}}_{j}(\tau )-{\text{net}}_{k}(\tau )}{2\gamma }\right)-w_{kj}(t)\right) & \text{if}\;\kappa (t)\neq \kappa (t-1)=k\\ 0 & \text{else} \end{array}\;,$$

where *T* denotes the number of time steps since the last winner transition, η_*l*_ denotes the learning rate for a lateral pattern weight, and κ(τ) denotes the winner pattern of time step τ. Batch-learning is particularly important in case of a small noise scaling γ, because otherwise the weight updates would be very close to either η_*l*_ or 0 in each time step, making it difficult to average over appropriate time spans.

#### 2.3.3. Self-supervision and backpropagation

By comparing the currently observed motion given by the concatenated populations' activation to the expected motion encoded by the currently recognized winner pattern, a prediction error can be derived without supervision. This error term δ_*i*_(*t*) is induced into the population neurons *i*, and is given by

(31) $$\delta_{i}(t)=w_{ik}(t)-o_{i}(t)\;,$$

where, *k* denotes the current winner in time step *t*. The error is top-down propagated along the feed-forward connections^4^ and finally merged at the perspective-taking module to adapt the orientation biases μ_*x*_, μ_*y*_, and μ_*z*_ in an error-minimizing manner (see Figure 1)—which is equivalent to a minimization of surprise, leading to a maximization of the current pattern's activation. Given the observed movement is similar to a rotated version of a movement that is encoded in the patterns, and given the correct motion pattern is recognized, the transformation to the closest perspective that was shown during the training is typically achieved. Thus, the model's ability to adapt its internal perspective in a self-supervised manner follows from the embodied encoding of biological motion via spatial and temporal associative learning, since the current winner pattern is determined both by the pattern best matching the stimulus and the expected sequence of patterns.

## 3. Experiments

In the following experiments, we show that (1) the model is able to encode a realistic walking movement when both visual and proprioceptive stimuli are present during self-perception; (2) multiple movements each in multiple frames of reference can be encoded in mainly disjunct sets of motion patterns, and (3) that this enables the transformation of randomly oriented views of similar biological motion to the previously learned frames of reference upon observation and thus the ability to solve the correspondence problem and to derive others' perspectives. To further evaluate the plausibility of the network in a neuro-cognitive context, we show that it is able to (4) reproduce psychological findings on bistable percepts of projected point-light walkers and (5) to simulate learned movements without sensory stimulation. Finally, we show that (6) the model is able to derive unobservable, hidden features such as the proprioception of another person when perceived from an unknown orientation. In this context, we point out that perspective-taking is necessary to facilitate this inference.

In all of the conducted experiments, we chose the following network parameterizations

$$\begin{array}{l} \text{ Input scaling}\;\alpha =5000\\ \text{ Smoothing factor}\;\lambda =0.95\\ \text{ Instar learning rate}\;\eta =0.01\\ \text{ Perspective-taking angular learning rate}\;\eta_{\mu }=0.0075\\ \text{ Perspective-taking angular momentum}\;m_{\mu }=0.85\\ \text{ Pattern recruitment threshold}\;\theta =\cos(60^{{}^{\circ}})\\ \text{ Pattern threshold breadth}\;b=0.034\\ \text{Pattern recruitment probability remainder}\;\epsilon =0.001\\ \text{ Lateral inhibition learning rate}\;\eta_{l}=0.6\;. \end{array}$$

In the following, we first introduce the simulation environment and stimuli we used to test the capabilities of our model and then proceed with the respective model evaluations.

### 3.1. Simulation environment and stimuli

We evaluate our model making use of motion tracking data recorded from three subjects, which performed three different, periodic movements (walking, running, and basketball dribbling) in four trials each. For each movement, we chose two of the trials as training set and the other two as testing set to test for generalization. Training, in this case, means enabling the segmentation and spatio-temporal learning of visuo-proprioceptive motion patterns while the network is driven by the training data sets to model mental development. Whereas testing means that the network is driven by the testing data sets while pattern learning is disabled to model and evaluate the model's action observation capabilities.

**Figure 6:**
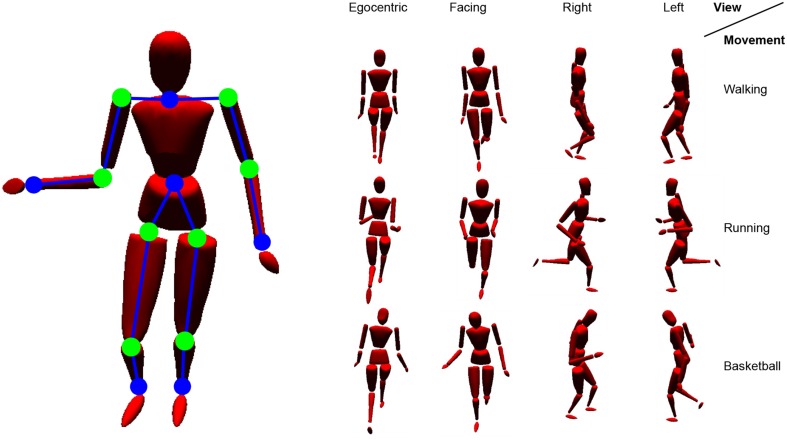
**Simulated body model driven by motion capturing data**. Shown are the relative visual features (blue lines between dots) and angles (green dots) chosen as input to the model. On the right, example stimuli are shown from different perspectives.

The motion tracking data were recorded at 120 Hz using 41 tracking markers attached to the subjects. As input to the model in all of the simulations, we chose the recorded time series of 12 three-dimensional, relative positions between the tracking markers as input to the visual pathway (each encoded by a 3D coordinate vector), as well as 8 angles between the relative positions as input to the proprioceptive pathway (each encoded by an angular scalar). In this configuration, the input layer of the network consists of 44 neurons. A map of the inputs at a single, exemplarily time step is given in Figure 6. Population coding of the individual features in Stage II results in a normalized, 348-dimensional common visuo-proprioceptive space in which motion patterns are put in place.

The motion tracking simulation we used provides positional information relative to any demanded frame of reference, including origin and orientation. In real life situations, specific vantage points are more common when observing biological motion. Considering an embodied framework, an egocentric perspective on the own motion will lead the way when learning biological motion. Such an egocentric perspective could be defined e.g., either head-centered or upper torso centered (Alsmith and Longo, 2014). Despite this embodiment, we assume that perspectives frequently perceived while observing others, or in relevant situations, may also have an influence on encodings of biological motion and account for the motion direction specificity observed in STS cells. Also, view-dependent encodings of actions may possibly emerge from social interaction and self-observation in mirrors (Rochat, 2003; Heyes, 2010). However, in the following experiments, we will particularly focus on four different (non-mirrored) perspectives: An *egocentric* view, which is learned first, as well as a *facing* view, a *right* view, and a *left* view, possibly resulting from pure observation or social interaction. Figure 6 shows some snapshots of recorded body motion from those different vantage points. Video examples of the stimuli are provided with the Supplementary Material.

Note that the origin of the coordinate system does not matter for the model and is thus not modified across the views. Because of this fundamental invariance, we are able consider a self-perceived movement visually equivalent to an observed movement of a distant person, as long as the orientation in space is the same. Also, the choice of the above perspectives is rather arbitrary for the learning algorithm itself. Here, we chose them since as a matter of principle (1) those views seem to be the most common and natural in social settings and (2) they divide the orientation space consistently about the vertical axis. Movements observed in other, rather uncommon orientations *not* encoded in the developing motion patterns are expected to be adapted to one of the learned views using the implemented perspective-taking principle.

### 3.2. Spatial and temporal motion pattern learning

In real life, learning about the own body is governed by an entangled nature of gathering knowledge and applying this knowledge. Newborns seem to be equipped with a to some degree developed body scheme of their proprioceptive and vestibular systems (e.g., Rochat et al., 1988). It is possible that components of those body schemes are even associated to respective visual stimuli later in life. However, we investigate in this first experiment, if our embodied model is principally able to develop a visuo-proprioceptive body schema from scratch during self-perception while its simulated body performs a walking movement, given that all information is available. That is, the developing pattern structure shall encode the own visual, relative body motion from an egocentric view as well as corresponding proprioceptive sensations that are characteristic for this kind of movement.

**Figure 7:**
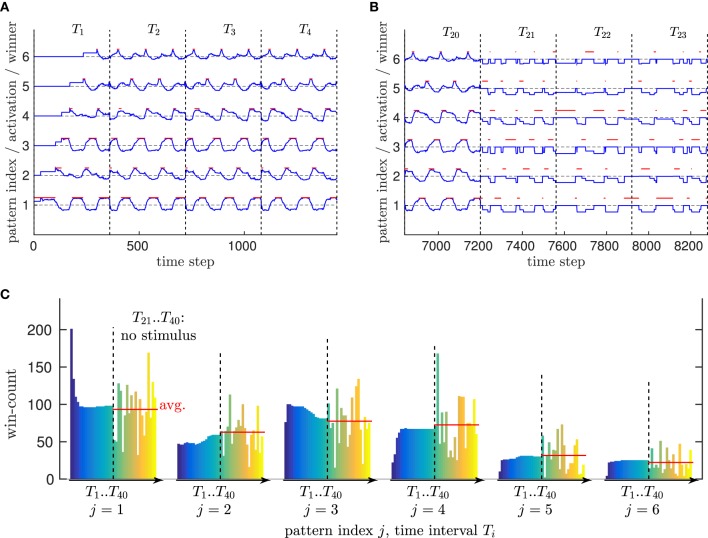
**Evaluation of spatial and temporal motion pattern learning. (A)** Pattern activation and winner patterns while a new movement is learned. Vertical bars restrict the time intervals *T_i_* in which a respective motion capturing trial is fed into the network. Blue indicates the activation of a pattern (dashed horizontal bars correspond to no activation), while red indicates which pattern is currently the winner pattern. Initially, pattern one is the only pattern in the network, which is converted from a free pattern to a trained pattern by adapting to the novel stimulus. Once this happens, a new free pattern (pattern 2) is created with activation ℂ(γ, θ). When the movement changes significantly, this pattern is also recruited. Because of the cyclic nature of the movement presented here, the series of winners is also cyclic. **(B)** Comparison of the pattern activation/winning cycle with (before time interval *T*_21_) and without sensory stimulus (starting with time interval *T*_21_). It can be seen that the same sequence of winner patterns (2 3 6 4 1 5) is activated in both cases repeatedly. This is a result of lateral probabilistic inhibition, where, for example, neuron 4 strongly inhibits all other neurons except its distinct follower 1. **(C)** Development of the winner histogram over time, when (time intervals *T*_1_ to *T*_20_) a movement is learned from an input stimlus, vs. the probabilistic sequence simulation when no stimulus is presented (time intervals *T*_21_ to *T*_40_). Each vertical bar counts the number of time steps neuron *i* is winning within the respective time interval. Without stimulus, a high variance of the winner histograms is the result of a the purely noise driven pattern neurons. However, the average winner histrogram seems comparable to the histograms determined after learning.

Thus, we drove the neural network by a single trial of the walking movement (performing about 6 walking steps in a 360 time steps interval *T_i_*), and repeated that training 20 times (*T*_1_..*T*_20_). Figure 7A shows how 6 patterns developed from scratch in their high-dimensional domain already during the first repetition *T*_1_, which then formed a cyclic series of winners because of the periodic nature of the walking movement.

To show that this pattern structure is stable over time and avoids a constant recoding of patterns as well as catastrophic interference (McCloskey and Cohen, 1989), we evaluated the number of time steps each of the patterns was winning (most active) during a repetition *T_i_* while the training of a motion tracking trial was continued—which we call the *winner pattern histogram*. Figure 7C shows that this histogram converged after roughly 16 repetitions.

Furthermore, we evaluated the temporal pattern sequence learning by clearing the activation of all population neurons (*g_n_*(*t*) = 0 ∀*n*, see Equation 19) after the training was finished, such that the pattern neurons were driven purely by noise and their lateral inhibition during the time intervals *T*_21_ to *T*_40_. Figure 7B shows that the same cyclic winner pattern sequence emerged despite the absence of sensory input, confirming the correct functionality of the temporal learning mechanism. Similarly, Figure 7C shows that on average the winner histograms of intervals *T*_21_ to *T*_40_ are comparable to the histograms of the training repetitions with stimulus *T*_1_ to *T*_20_. This indicates that the temporal pattern learning algorithm also approximates the correct movement speed without sensory stimulation.

These results confirm that the model is able to encode real-world biological motion patterns effectively: Neither does the network recruit unnecessary new patterns, nor are learned patterns or pattern sequences recoded constantly. Moreover, the systematic sequence of patterns in biological motion is learned correctly as well. The evaluation indicates that lateral inhibition can furthermore stabilize the recognition of motion pattern sequences, since unlikely pattern successors are inhibited in advance up to an equivalent of a 90° mismatch to the observed data.

### 3.3. Encoding multiple movements and perspectives

With respect to findings about view-based representations of diverse (also whole body) movements in STS (e.g., Oram and Perrett, 1996), we evaluate if the proposed model is also able to encode and differentiate a variety of different movements, each in different frames of reference. Thus, we selected the full training data-set—consisting of walking, running, and basketball movements—and trained them consecutively shown from the four perspectives defined in Section 3.1. We repeated this training procedure 20 times, resulting in 480 motion tracking trials presented to the network overall. Referring to selectivity of STS cells, this should result in 12 relatively independent groups of motion pattern neurons, each specifically responding to the view-dependent observation of a movement.

In a generic experiment, 151 patterns evolved during the training (± ~ 5% across independent experiments). After that, we drove the model by the testing data set, again showing four different orientations. We compared the winner histograms with respect to each of the 12 view-dependent movements, to see if patterns were responding exclusively and unambiguously to one of them, or if patterns were attributed to multiple view-dependent movements. Exclusiveness in pattern response decides whether the model can discriminate between movements and views. We assess that it can distinguish e.g., between the walking and the running movement perceived from the left view, or decide if someone is walking to the left or right if separable groups of motion patterns are activated during these observations.

**Figure 8:**
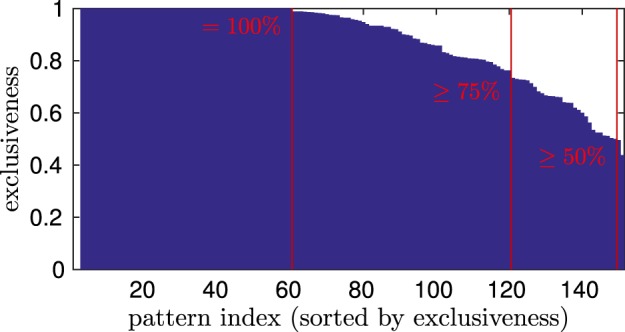
**Winning exclusiveness of the motion patterns with respect to 12 view-dependent movements**. The exclusiveness measure describes the maximum of the number of time steps a pattern neuron was winning while a specific view-dependent movement was observed, divided by the number of time steps the pattern was winning during the whole testing phase. Thus, 1 denotes the maximum exclusiveness, denoting exclusive winning during the observation of a specific movement and view, while the lower boundary is 1/12 for a pattern that is winning equally often during all observations.

Figure 8 shows the exclusiveness of pattern neurons after training. At first, it can be seen that 60 of 151 patterns were winning exclusively while a specific movement was shown from a specific vantage point, meaning an exclusiveness of 1. Further, 120 patterns were relatively unambiguously by representing over 75% of their winning time steps during the perception of a single view-dependent movement, implying a high affiliation. Finally, all except two patterns had a clearly relatable preference for a specific observation, meaning an exclusiveness of over 50%. This indicates that the motion patterns were encoded in 12 mostly disjunct sets with respect to the training data, although no supervised learning was applied. Thus, the model was able to separately encode, generalize, and recognize 12 different observations.

### 3.4. Perspective-taking

Based on the last experiment, where we could confirm that multiple movements and perspectives can be learned and recognized by the model, here we investigate the resulting perspective-taking ability and its precision when observing biological motion from vantage points not seen during the training. That is, when biological motion is observed in a random orientation, the model ought to minimize the divergence between the orientation the movement was encoded in, and the orientation it is observed in by adapting the bias neurons of the perspective-taking module in Stage Ic. Consider that in our model this perceptual adaptation is driven by an error signal provided by the currently recognized winner pattern. This can only work, however, when the sufficiently correct patterns are recognized, which can be compromised while the perspective is not properly adapted. In symbiosis, thus, perspective-taking will improve the probability to recognize the correct patterns, while correctly recognized patterns push the perspective adaptation further in the right direction. Upon convergence, we can evaluate the precision of the visuo-proprioceptive encodings as well as the robustness to variances in orientation, body morphology, and posture control by evaluating how precisely the perspective derived by the model matches one of the perspectives learned during the training.

Again, we first trained the network on 3 movements, each shown in 4 systematic perspectives as in the experiments before. After this, we drove the model by the complete testing set of the trained movements, consisting of 3 movements each performed by two different subjects. However, we applied a random rotation *R*_ν_ uniformly distributed in the 3D orientation space to the motion tracking data of each trial, and repeated its presentation until 5000 time steps were processed.

While doing so, we allowed the bias neurons μ_*x*_, μ_*y*_, and μ_*z*_ of the perspective-taking module in Stage Ic to adapt to minimize the top-down propagated error between the expected and the observed visual stimuli. We measured the difference between the resulting, internal rotation matrix *R*_μ_ applied by the model and the exogenous rotation *R*_ν_ applied to the simulation, with respect to each perspective that was trained on. Such a perspective can be defined by a rotation matrix *P_i_*, where *i* ϵ {1..4} depicts one of the four views defined in Section 3.1. This leads to a measuring unit *orientation difference* (OD) by calculating the trace of resulting overall rotation by

(32) $${\text{OD}}_{i}(t)=\;1/2\cdot \text{acos}(\text{tr}(P_{i}^{\prime }R_{\nu }R_{\mu }(t))-1)\;,$$

which describes the minimal amount of rotation about an arbitrary 3D axis to transform the currently derived orientation *R*_μ_*R*_ν_ to the encoded orientation *P_i_*. That is, *OD_i_*(*t*) converges to 0° when the model internally compensated the rotation applied to the simulation. In this case, the unknown, observed perspective has been adopted and the correspondence to the learned encodings has been established.

We ran this experiment 500 times, including independent training with different random seeds. Regardless of the exogenous orientation *R*_ν_, over 97% of the shown movements could be transformed to one of the learned views *P_i_*. The orientation of an observed movement was considered successfully derived, when the OD converged to less than 35° after 5000 time steps. While there is a clear preference for convergence to the perspective that is nearest in terms of the OD, the model does not feature a strong preference for a specific perspective *P_i_*, such that all of the perspectives were reached about equally often. Thus, the model is able to derive the perspective of another person performing a movement similar to a movement known from self-perception on the fly, without explicit knowledge about their orientation, and by motion signals only. In informal tests, we were not able to reproduce the same success rates when no temporal pattern learning was applied.

**Figure 9:**
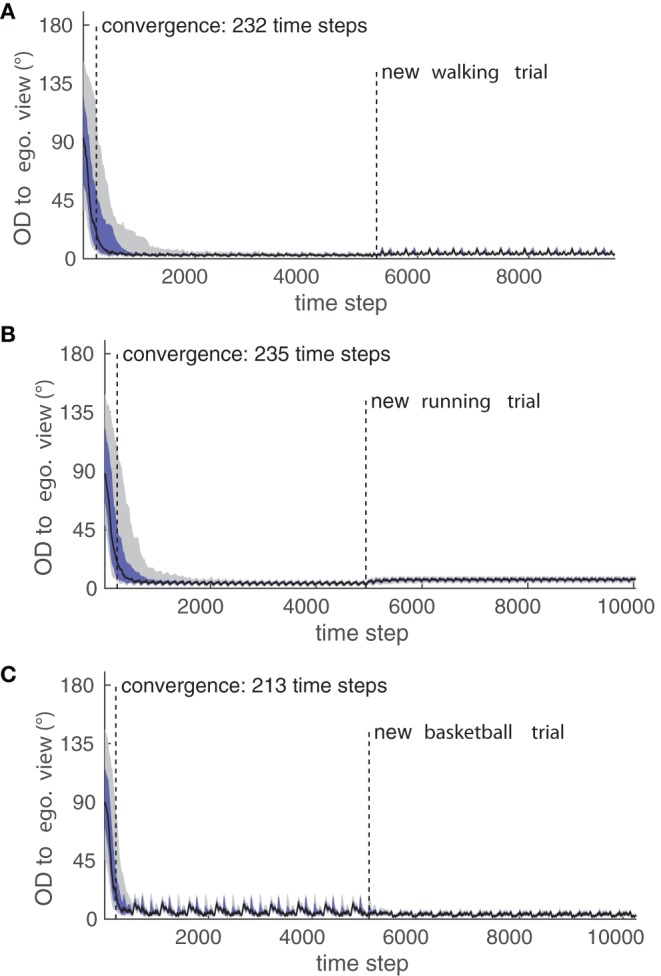
**Perspective-taking experiment**. Shown is the convergence of the Orientation Difference (OD) to the egocentric view for (A) the walking movement, (B) the running movement, and (C) the basketball movement while the testing trials were shown. Black indicates the median of all trials, dark blue indicates the 25/75% quartiles, and gray indicates the 10/90% deciles of the OD. The plots for the convergence to the other three learned views are similar respectively, and their convergence properties can be see in Table 1.

Figure 9 shows the convergence to the egocentric view *P*_1_ in terms of OD over time for all examined movements and trials in the testing data set. Particularly, it can be seen that different trials of the same movement result in differing variances in the final OD after convergence, which was raised by deviations in posture control to the training trials. Since the model applies normalization to each considered feature, different body morphologies are generalized over and can not be a source for such a variance in OD. Rather, more complex and articulated movements are more likely to have a higher degree of postural control variance over several trials, which explains the high difference in the remaining OD between the two tested basketball trials.

The convergence properties of all experiments are detailed in Table 1: While the convergence time was comparable in all cases, it can be seen that the relatively fast running movement in comparison to the walking is conspicuous by a slightly worse precision in terms of the remaining OD after convergence on average over both testing trials. Also, the more complex basketball dribbling shows a final variance in the median OD larger than the two other motion types.

**Table 1:** **Overview of the convergence properties in the perspective-taking experiment**.

**Movement**	**Perspective**		**Convergence (%)**	**Convergence time**	**Remaining OD (°)**	**Median variance (°)**
Walking	Egocentric	:	21.4	232	3.42	0.89
	Right	:	27.4	215	3.32	0.91
	Left	:	22.6	218	3.32	0.84
	Facing	:	23.8	257	3.42	1
	∑ or ∅		95.2	230.5	3.37	0.91
Running	Egocentric	:	22.2	235	5.32	2.34
	Right	:	24.6	240	6.1	2.52
	Left	:	26.2	206	5.46	2.41
	Facing	:	23.4	251	5.71	2.2
	∑ or ∅		96.4	233	5.65	2.37
Basketball	Egocentric	:	25.8	213	4.56	3.99
	Right	:	23.2	229	4.78	6.57
	Left	:	28.6	230	4.41	4.55
	Facing	:	22	243	4.45	3.37
	∑ or ∅		99.6	228.75	4.55	4.62

*When testing, we measured the percentage of runs that converged to each specific perspective (Convergence). Further, we evaluated the time step the median of the OD fell below 20° (Convergence time). The Remaining OD denotes the average of the median OD at the end of both trials shown, while Median variance depicts its variance*.

### 3.5. Bistable stimulus

To further evaluate the plausibility as a neuro-cognitive model of brain functionality, we also investigated bistable properties of the network: It has been shown that humans recognize biological motion perceived from point-light displays with specific orientations bi-stably, either as pointing away (corresponding to our egocentric view) or toward the viewer (corresponding to our facing view) when the walker was perfectly symmetric and projected on a 2D screen (albeit with preference to the facing view) (Vanrie et al., 2004).

**Figure 10:**
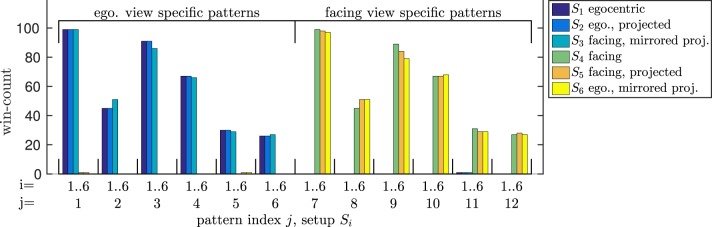
**Bistable stimulus experiment**. According to the activated patterns, all egocentric and facing views of the walking movement as well as their 2D projections are recognized correctly. However, when the movement is projected and the display is inverted about the vertical axis, egocentric views are recognized as facing and vice versa.

Thus, we trained the model both on the egocentric and the facing view of the walking movement. After training, we evaluated the influence of a parallel projection to 2D, as well as the influence of gait symmetry on the correct recognition while the same data was presented again. The pattern winner histograms with respect to all possible setups (*S*_1_..*S*_6_) in Figure 10 first show that the model was able to distinguish the 3D representations of the facing and egocentric walking movements by means of exclusively winning patterns. More surprisingly, the model was also able to recognize the walking direction correctly when projected to 2D. Further investigation showed that this is a result of the asymmetry of the movement that was trained on: By inverting the horizontal component of the movement, we achieved the opposite effect: The facing view was recognized as egocentric and vice versa.

While we could thereby replicate a bistable perception, we could also ensure that the model is able to recognize biological motion that was learned in a 3D visual domain from a 2D projection. Thus, we suggest that the model will also work robust on data with uncertain depth component.

### 3.6. Feature inference

In this final experiment, we ensure that information that was trained by self-perception, but is not available during the observation of another person, can be derived by activating the correct motion patterns even when observed from rather uncommon perspectives.

We trained the network on a walking movement perceived from an egocentric view as in experiment 3.2, such that pattern neurons were encoding visual and proprioceptive stimuli. When testing the movement and enabling the network's perspective-taking, we compared (1) the prediction error in the proprioceptive population neurons while pattern neurons were driven both by vision and a proprioceptive equivalent^5^ during the observation, with (2) the prediction error that was measured while patterns were driven by vision only. Thereby, we investigate if the model is nevertheless able to recognize the correct visuo-proprioceptive motion patterns and thus inferres the missing proprioception. Also, we investigate, if missing information impairs the ability adapt to perspectives: In both cases (1) and (2), we rotated the visual representation of the walker by 180° about the walking direction (leading to top-down inversion), followed by 45° about the vertical axis after learning was complete, which amounts to an almost-worst-case scenario in terms of orientation difference to the encoded biological motion.

**Figure 11:**
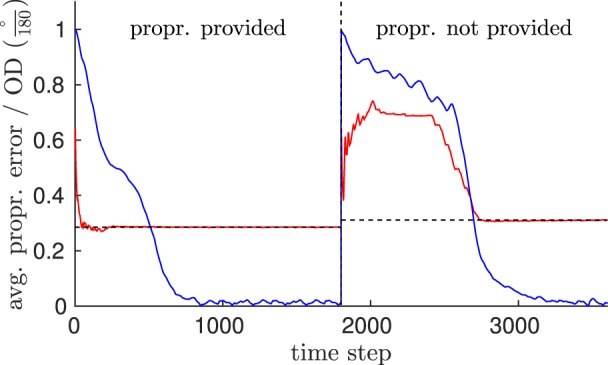
**Evaluation of the model's feature inference capability**. This shows a comparison of the prediction error in the proprioceptive pathway (RMS) while pattern neurons were driven by both visual and proprioceptive information (left side of the dashed vertical line), in contrast to the error observed while pattern neurons were driven by vision only (right side of the vertical line). Information was given by the observation of a walking trial, perceived from an unknown view. Blue indicates the orientation difference of the derived orientation to the egocentric orientation, while red indicates the moving average of the error in inferring proprioception (dashed horizontal lines indicate the level of convergence).

Figure 11 shows that the prediction error in the proprioceptive pathway started relatively high in both cases, suggesting incorrectly recognized patterns. That is, without adoption of the perspective, the proprioception expected by the model was faulty. However, the error converged to a final level of ~0.28 (1) or ~0.31 (2), respectively as the adaptation of the perspective progresses, regardless whether the proprioception was provided or not. When patterns were driven purely by vision, the performance of this adaptation was compromised. However, the final error was comparable, confirming that the proprioceptive information was inferred precisely as well after the perspective was derived.

Substantially, this result suggests that visuo-spatial perspective-taking can be considered a candidate for solving the correspondence problem: Since biological motion seems to be encoded view-dependently, similar perceptual adaptation mechanisms appear necessary to explain action understanding and inference abilities in the human brain.

Analogously to the derivation of the proprioception by vision, the model can also infer the correct visual motion patterns when it is driven by proprioceptive information only. However, this process does not require perceptual adaptation. Another result is, that adding orientation independent, angular information as visual equivalent to proprioception can help to recognize the correct patterns and speed up the derivation of the perspective and potentially other intrinsic states.

## 4. Related work

There is a number of noteworthy related approaches. Amongst them, Fleischer et al. modeled the properties of abstract STS cells during object interaction. Their approach includes the encoding of multiple viewer-centered representations of simple, schematic actions to establish a certain degree of orientation invariance. The recognition was based on a hierarchy of feature detectors in several neurobiologically inspired domains, like local shape detectors and motion neurons, leading to plausible model predictions about the human recognition performance (Fleischer et al., 2013). However, using separate networks for each encoded view-point seems counterintuitive. Also, the model uses a hard-coded wiring and parameterizations that is not trained on data.

Lange et al. modeled biological motion recognition using viewer-centered, image-based posture templates, where the best matching template responses were integrated over time and decided on the recognized movement (Lange and Lappe, 2006; Lange et al., 2006). This model is timescale-independent to a certain degree and can distinguish pre-defined walking directions. The approach also produced plausible results with respect to the artificial cell firing rates. Even so, the model is working on a domain where scale- and translation invariance are assumed. Further, the motion information is only considered indirectly by recognizing whole movements by means of adjacent posture images, and the model was validated only on a single movement in two manually distinguished orientations. Again, no learning was applied to the model's parameters.

Schindler and Van Gool were able to show that a model that processes both form and motion information is able to recognize and distinguish several actions from very short motion clips (Schindler and Van Gool, 2008). Although this result is admirable, their approach is again based on pre-parameterized local template matchers, max-pooling operators and supervised, linear classification methods. Further, it does not provide any explicit mechanism to achieve spatial or temporal invariances.

A neural network related to our approach modeling STS cells for biological motion perception was developed by Layher et al. (2014). The model includes neurally plausible Hebb'ian learning mechanisms to integrate form and motion pathways and identify relevant postural snapshots of biological motion. Also, the model includes top-down signal processing that reinforces the encoding of articulated postures. As a result, walking movements could be learned, recognized and distinguished with respect to the walking direction without supervision.

All of the work mentioned above has in common, that biological motions and actions are neither encoded view-invariantly, nor is invariance—especially to orientation—established on-line to a plausible degree. In contrast, our approach gains a high degree of spatio-temporal invariances merely due to adequate neural processing and encoding, and attains orientation invariance by converging to perceptual attractor states. To the best of our knowledge, this ability is a unique property of our model, which allows it to form a bridge between visual perceptions and corresponding perceptions in other modalities.

Also, our model's ability to distinguish specific viewer-centered movements is not based on pre-wired templates or supervised classification, as in all of the foregoing models except Layher et al. (2014). In contrast, we apply unsupervised spatio-temporal clustering paradigms that facilitate perceptual flexibility: Top-down propagation of a self-supervised prediction error consequently allows adaptation of the visual perception. This is equivalent to a predictive-coding scheme (Rao and Ballard, 1999; Friston et al., 2006) minimizing the free energy emerging from experiencing surprise about the directionalities in biological motion observed from an unknown vantage point. Also, this parallels the direct inference of the spatial orientation of another person merely by inspecting relative motion signals.

However, to make the model sparse and its adaptation ability computationally efficient (and to avoid sliding into the topic of image-processing and feature extraction), we abstract the retinotopic and tuning properties of occipital/temporo-occipital and parietal visual processing sites by feeding the network directly with analytically relevant information about biological motion. This allows us to verify the model on realistic and complex 3D scenarios in real-time. Whereas the above models thus mainly rely on a combination of form, shape and partially motion template snippets, our model solely operates on abstract relative motion signals as the probably most essential domain for the recognition of actions. This is in accordance with the fact that local motion features are most critical and necessary for perceiving biological motion from point light displays (Johansson, 1973; Garcia and Grossman, 2008; Thurman and Grossman, 2008).

Further, our model does not solely work with visual features. To give our model basic mirror neuron properties, we include motor-related proprioceptive codes in our model. This allows the derivation of intrinsic, otherwise unobservable states during the observation of others. Under the assumption that proprioceptive information can partially be derived directly from vision, this also increases the recognition robustness of the model.

Previously, we could show that using an embodied, generative model on a minimal set of abstract, relative, visual and proprioceptive motion information, it is possible to transform observed 2D biological motion to canonical frames of reference (Schrodt et al., 2014a). The model also adopts to simulated full body motion, whereas the predictive coding scheme provides a high precision and recognition performance of sufficiently upright walkers even in 3D spaces (Schrodt et al., 2014b). Adding a simple algorithm that forecasts the sequence of observed motion patterns can ensure the recognition of movements shown in arbitrary, also top-down inverted perspectives. Beyond the mentioned spatio-temporal robustness, the model is completely invariant to body morphology and invariant to variabilities in posture control to a certain degree (Schrodt and Butz, 2014).

In this work, we applied the model to complex motion-tracking data and provided a more elaborate spatio-temporal pattern learning algorithm that is able to encode ambiguous sequences of motion, provides the possibility to simulate movements even in the absence of sensory stimulation, and advances the unsupervised distinction of observed movements and views. Also, we have shown the necessity of perspective-taking for the derivation of others' intrinsic bodily states (e.g., joint angles), given that visual encodings of biological motion are represented view-dependently.

## 5. Discussion

The presented modeling results have shown that the introduced generative, neural network model learns to encode biological motion, enabling the invariant and robust recognition of observed movements and adoption of others' perspectives. The neural noise based pattern learning paradigm has proven to be suitable for both learning spatial and temporal multimodal correlations: The emerging sets of patterns encoding view-dependent movements were predominantly disjunct and classifiable without any form of supervised learning. The motion patterns essentially provided self-supervised signals to adapt an internal visual perspective online while preserving a high degree of robustness to realistic variances in observed movements. The temporal pattern learning algorithm that improves the recognition performance when biological motion is observed was capable of simulating whole movements probabilistically when no sensory stimulation was present. Psychological findings on bistable percepts of biological motion could be replicated in the experiments, which underlines the plausibility of our network in a neuro-cognitive context. Finally, we were able to show that others' intrinsic states can be inferred solely by observing visual bodily motion signals under the assumption of an embodied learning framework.

Our experiments clarify that perspective-taking is a prerequisite in this process when biological motion is encoded view-dependently but observed from rather unknown perspectives. Thus, hypothetical concepts attempting to explain the mirror neuron property to derive action related codes from observation should consider similar spatial visualization abilities as a potential solution to the correspondence problem. Complementary to the ideomotor theory, the associative sequence learning hypothesis states that somatosensory and motor representations of actions are associated to their visual equivalent while perceiving the own actions, but also while being imitated, perceiving a mirrored self, as well as synchronous activities with others (Heyes and Ray, 2000; Heyes, 2001; Brass and Heyes, 2005; Heyes, 2010). This would lead to the ability to infer action related codes during the observation of others to enable imitation. However, our results suggest that the correspondence problem cannot be solved reliably by a generalist, solely associative model, as opposed to the claims of the authors. We believe that perspective-taking can help the activation of view-dependent representations of actions and the derivation of others' intrinsic states by establishing the correspondence between frames of reference. Analogous concepts could thereby explain social abilities related to the mirror neuron system at a tangible level of detail.

Furthermore, our modeling paradigms suggest a functional benefit of neural noise: Besides breaking the symmetry in uninitialized or equal encodings, it allows to implicitly encode stochastic activation sequences in laterally connected clusters of cells. Reciprocal inhibition in combination with neural noise could thus explain neuronal avalanches observed in cortical circuits (Beggs and Plenz, 2003, 2004).

Future validations of the model should compare the orientation specificity in the model's ability to recognize biological motion from point-light displays with the performance of humans (Pavlova and Sokolov, 2000). We further anticipate that our embodied model can be used to identify sex (Runeson and Frykholm, 1983) or even the identity (Cutting and Kozlowski, 1977) of an observed person solely by motion signals, and performs better in the latter task when observing recordings of the own actions from point-light displays in comparison to the actions of others (Beardsworth and Buckner, 1981).

There are certainly several limitations in our current model. First of all, it relies on motion signals only, such that it has no possibility to adopt the perspective of another person simply by observing their posture. By adding postural information to the model, its ability to derive others' perspectives could be improved. Also, further intrinsic modalities should be included in the embodied learning procedure. Our model is able to expect and infer unobservable proprioceptive features to a certain extent. Analogous approaches could enable the derivation of further states, like executed motor primitives or simulated action intentions, which could drive forth a realistic model of self-supervised learning by imitation.

Currently, a major limitation of our model is the fact that we indirectly supply a basic body-structure knowledge to the model by manually selecting and assigning bodily features to specific network inputs. That is, we define that e.g., the first visual input to the model is responsible for processing the relative location of the elbow. For a biological system observing a point-light display this assignment is a non-trivial problem since it isn't supplied with body-structure information a priori. Thus, in our current research we focus on mechanisms that automatically and dynamically select features and assign them adequately to the respective neural processing pathway during the observation of motion. First investigations showed that the same prediction error signal that is used for perspective-taking can also be used for this task.

The introduced spatio-temporal pattern learning algorithm currently relies on angular distances between activations given by a set of neural populations. However, in terms of an angular distance metric between different stimuli, coding neural activation in symmetric populations is equivalent to coding them in directional vectors. Except to account for small motion signals, it is thus not necessary to convert the neural coding paradigm to population coding in the current version of our model. However, populations provide the potential to encode multiple, possibly conflicting stimuli in the same population. In the context of biological motion recognition from point light displays, the model could be equipped with mechanisms that maintain multiple feature selections in parallel, leading to uncertain stimulus encodings until a perceptual attractor state is reached. Moreover, incertainty could be expressed by adapting the noise scaling parameter γ. An adaptation of the pattern recruitment threshold θ could account for an increase in spatial resolution in crucial parts of observed movements, whereas distinct normalization lengths' per feature could be used to express the relevance of specific features.

Despite the high invariance of our model to several spatio-temporal transformations, the invariance to speed and scale is limited by the fixed scaling parameter α to a small degree, when no velocity is present in some of the observed bodily features. In future model versions, this scale should be adapted online analogously to the orientation of the frame of reference, effectively implementing both a temporal and spatial zooming mechanism.

### Conflict of interest statement

The authors declare that the research was conducted in the absence of any commercial or financial relationships that could be construed as a potential conflict of interest.

## Footnotes

^1^The model can thus also process, but does not necessarily rely on joint angles.

^2^For an one-dimensional angular feature, this means that only the signum of the angular change is considered.

^3^Analogously, the rotation could be described by any possible extrinsic or intrinsic Euler or Tait–Bryan rotation sequence, because the self-supervised adaptation mechanism accounts for the correctness of the resulting axis, direction, and degree of rotation.

^4^This means that all presynaptic process functions implicitly implement backpropagation functions for feedback processing.

^5^Here, we assume that proprioception can partially be derived directly from vision.
