# Supplementary material for: Embodied learning of a generative neural model for biological motion perception and inference
Source: Front Comput Neurosci. 2015 Jul 6;9:79. doi: 10.3389/fncom.2015.00079 (PMC4491628; doi:10.3389/fncom.2015.00079)
Supplement: Supplementary file 14 [file SupplementaryMaterial_Derivations.PDF]

Frontiers Computational Neuroscience

Original Research
201524 June 2015
10.3389/fncom.2015.00079

Supplementary Material: Embodied Learning of a Generative Neural Model for Biological Motion Perception and Inference

Fabian Schrodt [1,*], Georg Layher [2], Heiko Neumann [2] and Martin V. Butz [1]

[1] *Department of Computer Science, University of Tübingen, Tübingen, Germany*
[2] *Institute of Neural Information Processing, Ulm University, Ulm, Germany*

Correspondence*:
Fabian Schrodt
University of Tuebingen, Department of Computer Science, Cognitive Modeling, Sand 14, 72070 Tuebingen, Germany, tobias-fabian.schrodt@uni-tuebingen.de

SUPPLEMENTARY VIDEOS

Videos of biological motion stimuli used in the experiments are available at:

http://journal.frontiersin.org/article/10.3389/fncom.2015.00079/abstract

SUPPLEMENTARY DERIVATIONS

PATTERN RECRUITMENT PROBABILITY

Given the activations $o_f(t)$ and $o_g(t)$ of two pattern neurons, the probability that the activation of pattern f is greater than the activation of neuron g follows from the cumulative distribution function of the difference of two Cauchy-distributed random variables:

$$\begin{aligned} o_g(t) &= \mathbb{C}(\gamma, \mathrm{net}_g(t)) \ , \\ o_f(t) &= \mathbb{C}(\gamma, \mathrm{net}_f(t)) \ , \\ \Rightarrow p(o_f(t) \geq o_g(t)) &= p(o_g(t) - o_f(t) \leq 0) \ , \\ &= \mathbb{CC}(0, 2\gamma, \mathrm{net}_g(t) - \mathrm{net}_f(t)) \ , \end{aligned} \tag{1}$$

where $\mathbb{CC}(a, b, c)$ denotes the cumulative probability distribution that a Cauchy-distributed random variable with scaling b and mean c is less than or equal to a. Given that pattern g is the best matching trained pattern and pattern f is the free pattern with

$$\mathrm{net}_f(t) = \theta \ ,$$

the probability that the free pattern is the winner and is thus recruited to represent the current driving data results in

$$\boxed{p(o_f(t) \geq o_g(t) \mid \mathrm{net}_f(t) = \theta) = 1/2 + 1/\pi \cdot \arctan\left(\frac{\theta - \mathrm{net}_g(t)}{2\gamma}\right)} \ .$$

PATTERN NEURON NOISE PARAMETRIZATION

Given the probability ϵ that a new pattern f is recruited while the best matching trained pattern g has net input $\theta + b$:

$$p(o_f(t) \geq o_g(t) \mid \text{net}_g(t) = \theta + b) = \epsilon$$

the parameter γ is determined by ϵ and b:

$$\begin{aligned}
&\overset{(1)}{\Rightarrow} \mathbb{CC}(0, 2\gamma, \theta + b - \theta) = \epsilon \\
&\Rightarrow {}^1\!/_2 + {}^1\!/_\pi \cdot \arctan\left(\frac{-b}{2\gamma}\right) = \epsilon \\
&\Leftrightarrow {}^\pi\!/_2 + \arctan\left(\frac{-b}{2\gamma}\right) = \epsilon\pi \\
&\Leftrightarrow \frac{2\gamma}{b} = \tan(\epsilon\pi) \\
&\boxed{\Leftrightarrow \gamma = \frac{\tan(\epsilon\pi) \cdot b}{2}}
\end{aligned}$$

LATERAL INHIBITORY PRE-SYNAPTIC PROCESS FUNCTION

Given that the output of a pattern neuron j

$$o_j(t) = \mathbb{C}(\gamma, \text{net}_j(t))$$

is determined by signal $\sum_i s_{ij}(t)$, noise $\mathbb{C}(\gamma, 0)$, and a lateral inhibition $s_{kj}(t)$ by another pattern neuron $k \neq j$, such that

$$o_j(t) = \mathbb{C}(\gamma, 0) + s_{kj}(t) + \sum_i s_{ij}(t) \quad ,$$

while the output of the pattern neuron k is determined analogously, but without lateral inhibition. Given also the probability $p(o_j \geq o_k) = w_{kj}$ that the activation of neuron j is greater than the activation of neuron k, it follows by Equation 1 that

$$\begin{aligned}
w_{kj} &= {}^1\!/_2 + {}^1\!/_\pi \cdot \arctan\left(\frac{[s_{kj}(t) + \sum_i s_{ij}(t)] - [\sum_i s_{ik}(t)]}{2\gamma}\right) \\
\Leftrightarrow w_{kj}\pi &= {}^\pi\!/_2 + \arctan\left(\frac{s_{kj}(t) + \sum_i (s_{ij}(t) - s_{ik}(t))}{2\gamma}\right) \\
\Leftrightarrow \tan(w_{kj}\pi) &= \frac{-2\gamma}{s_{kj}(t) + \sum_i (s_{ij}(t) - s_{ik}(t))} \\
\Leftrightarrow s_{kj}(t) + \sum_i (s_{ij}(t) - s_{ik}(t)) &= \frac{-2\gamma}{\tan(w_{kj}\pi)} \\
\Leftrightarrow s_{kj}(t) &= \frac{-2\gamma}{\tan(w_{kj}\pi)} + \sum_i (s_{ik}(t) - s_{ij}(t)) \quad .
\end{aligned}$$

Under the assumption that there is no signal, and by applying a hyperbolic tangens to limit the range of the lateral inhibition, we approximate the lateral inhibitory pre-synaptic process function by

$$\boxed{s_{kj}(t) \approx \tanh\left(\frac{-2\gamma}{\tan(w_{kj}\pi)}\right)}$$
